# Supplementary material for: Are carbon emissions trading and green financial instruments synergistic? -Comprehensive quantitative research based on content analysis
Source: PLoS One. 2024 Mar 7;19(3):e0298601. doi: 10.1371/journal.pone.0298601 (PMC10919601; doi:10.1371/journal.pone.0298601)
Supplement: S1 Data — (ZIP) [file pone.0298601.s001.zip › Data/地级市Z_F工作报告.zip!/╒π╜¡/└÷╦«╩╨/└┤╫╘╣1⁄2╓┌║┼í╛┬φ┐╦╩2╛▌═°í┐_2017.docx]

各位代表：

　　现在，我代表丽水市人民政府，向大会报告政府工作，请予审议，并请市政协委员和其他列席人员提出意见。

　　一、过去五年回顾和2016年主要工作

　　市三届人大一次会议以来的五年，是丽水发展抢抓机遇、砥砺奋进的五年。在省委、省政府和市委坚强领导下，全市上下深入学习贯彻习近平总书记治国理政新理念新思想新战略，以省委“八八战略”为总纲，扎实推进“绿水青山就是金山银山”战略指导思想落地生根，迈出了建设美丽幸福新丽水的坚实步伐。

　　——区域综合实力和生态环境质量实现“双提升”。全市生产总值从2011年的798.83亿元增加到1200.22亿元，年均增长8.0%，分别高于全国和全省0.7个、0.1个百分点；人均生产总值达到55772元。财政总收入、一般公共预算收入分别达164.87亿元、103.57亿元，年均分别增长10.5%、12.5%。发展水平指数全省第三，发展进程指数全省第一，各县（市、区）均摘掉欠发达帽子。与此同时，生态环境状况指数连续13年全省第一，生态文明总指数跃居全省第一；被列为全国生态文明先行示范区、生态保护和建设示范区、水生态文明试点城市，成为首个地级市“中国长寿之乡”、唯一的“中国气候养生之乡”；实现省级生态县、省级森林城市全覆盖，云和、遂昌、庆元、缙云创成国家生态县。

　　——发展方式转变和发展动能转换实现“双推进”。三次产业结构由9.1‥49.8‥41.1调整为8.0‥44.7‥47.3。生态工业转型向好，工业技改投资年均增长37.9%，规上工业新产品产值率达到35.6%，高新技术产业增加值占规上工业增加值比重达到27.7%，丽水开发区升格为国家级经济技术开发区。创建了覆盖全区域、全产业、全品类的生态农业区域公用品牌——“丽水山耕”，获得休闲农业与乡村旅游“国字号”头衔23个，农业标准化覆盖面达64.4%。成为国家全域旅游示范区首批创建单位，旅游总收入连年保持25%以上高速增长，文化产业增加值占GDP比重从全省末位跃居全省第三。改革、创新、开放形成的新动能加速集聚，尤其是以林权改革为先导的农村产权及农村金融、扶贫、电商等方面改革试点赢得广泛关注，打造了“丽水样本”，农村金改“丽水模式”成为“全国十大自下而上的金融改革之一”；首次荣获全国县（市）科技进步考核先进市，被列为创建国家知识产权试点城市；累计实际利用内资、外资分别达1338.7亿元、8.5亿美元，引进已落地大项目583个，净增市场主体9.8万户，民间投资年均增长22.8%，出口年均增长11.7%。

　　——城乡基础设施和城乡社会面貌实现“双优化”。累计完成固定资产投资3300.65亿元、年均增长21.1%，其中基础设施投资1346.22亿元、年均增长22.3%。建成云景、龙庆、龙浦高速，实现“县县通高速”。完成金丽温铁路扩能改造工程，迈入“高铁时代”。常住人口城市化率达58.0%，中心城市建成区面积达35平方公里，建成区绿化覆盖率、绿地率分别达44.8%和40.4%，开发利用地下空间163.5万平方米。成功创建国家森林城市、园林城市、卫生城市和省级历史文化名城。全市实现公交车和公共自行车IC卡互联互通，市区成为“长三角十大公共自行车推广典型城市”，新能源汽车分时租赁系统快速铺开。通过“五水共治”，全面消除垃圾河、黑臭河，新增污水管网772.35公里，完成农村生活污水治理2329个村、受益农户40万户。通过“三改一拆”，累计拆除违法建筑2782.8万平方米，完成“三改”4475.81万平方米，完成市区26个老小区（城中村）的改造提升。通过美丽城乡建设，基本形成“一县一主题、一县一特色”县域城市风貌，158个村入选中国传统村落保护名录、8个村入选中国少数民族特色村寨，我市成为“中国美丽乡村建设示范地区”。

　　——人民生活水平和基本公共服务实现“双改善”。全市财政用于民生支出五年累计达908.33亿元，财政民生支出比重从70.1%提高到77.6%。城乡居民人均可支配收入年均分别增长9.8%、11.7%，农民人均可支配收入达到16459元，超出全国平均水平4096元，增幅连续8年居全省首位，完成“4600元以下”消除贫困任务。就业持续扩大，基本建成统筹城乡、惠及全民的社保体系，居家养老服务照料中心覆盖100%的城市社区和92.4%的农村社区，各类保障性安居工程住房受益群众25.67万人，农民异地搬迁9.55万人。11个民族村成为省民族团结进步小康村，所有民族村均通康庄公路。实现国家义务教育基本均衡县全覆盖，省义务教育标准化学校比例从11.6%提高到89.3%，丽水学院通过本科教学工作审核评估。“乡村春晚”获国家公共文化服务体系示范项目创建资格，文化发展指数稳居全省前列。入选全国十佳运动休闲城市，实现省级体育强县全覆盖。建成市博物馆、美术馆、体育馆。医疗服务达到全省中等水平，人均期望寿命提高到79.79岁。残疾人“两项补贴”制度惠及73.6%的持证残疾人。老龄、妇女、儿童、慈善、宗教和对台、侨务等工作得到加强。

　　——社会治理创新和社会和谐稳定实现“双加强”。连续8年被评为全省法治政府建设优秀市，3个典型案例先后入选年度“中国全面小康十大社会治理创新奖”。深入推进“四张清单一张网”改革，非行政许可审批事项全面清零，居民个人审批服务事项即办率达86.3%。创新实施“平安报表”管理法，连续12年被命名为平安市，治安案件、“两抢”案件连续5年下降，各类事故起数、死亡人数连续5年“零增长”。实现创建全国双拥模范城“两连冠”。全市上下众志成城，先后战胜“8·20”全流域特大洪灾、“11·13”里东山体滑坡、“9·28”苏村山体滑坡等突如其来的重大灾害，所展现的新时期丽水精神，已成为激励全市上下奋发进取的强大正能量！ (下转第五版)

　　(上接第一版）

　　五年来，我们还积极支持各县（市、区）立足全市“一盘棋”、走好“特色路”：莲都被确定为国家农业综合开发现代农业园区试点；龙泉青瓷、宝剑产业被列入省十大历史经典产业；青田跻身全国县域经济竞争力和发展潜力“双百强”；云和成为国家中小城市综合改革试点县、“浙江美丽县城”首批试点县；庆元获评中国绿色竞争力最强县；缙云培育了“一个烧饼、三个家家”农民增收品牌、成为省级综合性“腾笼换鸟”试点单位；遂昌入选全国首批旅游标准化示范县、孕育了电商“赶街”模式；松阳被列为中国传统村落保护利用试验区；景宁成为全国农村产权流转交易标准化试点县、中国最佳民族风情旅游名县。所有这些，都是打开“两山”转化通道的生动实践！

　　刚刚过去的2016年，是“十三五”开局之年。我们牢牢把握“绿色发展、科学赶超、生态惠民”发展主线，归零翻篇开新局，精准发力补短板，实现了开局之年全年红。年初提出的主要预期发展指标，除农民人均可支配收入增长9.7%，增幅虽保持全省第一，与预期的10%略有差距外，其余均达到或超额完成目标，十方面民生实事也件件落地。重点抓了以下工作：

　　（一）打好强生态促保护组合拳。积极探索生态法治，完成《丽水市城市市容和环境卫生管理条例》立法，完成自然资源资产负债表编制省级试点，制定了党政领导干部生态环境损害责任追究实施细则。组织开展“7?29”首个丽水生态文明日系列活动。全面划定永久基本农田。5县（市）入选新增国家重点生态功能区。勇夺“五水共治”大禹鼎，腊口污水处理厂开工建设，新增污水管网180.04公里，新建供水管网115.2公里、改造供水管网117.3公里，完成河道综合整治253.84公里，饮用水源地、跨行政区域河流交接断面水质达标率均为100%，水环境质量全省第一。全面开展“五气共治”，淘汰改造燃煤锅（窑）炉427台，淘汰黄标车和老旧车5789辆，各县（市、区）PM2.5浓度平均值均低于35微克/立方米，市区空气质量优良天数从上年的327天增加到349天，优良天数全省最高。

　　（二）打好强产业促转型组合拳。结合丽水实际抓好“三去一降一补”，坚决淘汰落后产能，商品房去化周期保持合理水平，金融机构不良贷款余额、不良贷款率实现双降，多措并举减轻企业负担30.97亿元。着眼于补好生态产业发展短板，出台各类产业政策14个，签订各类战略合作协议15个，设立了大健康产业、绿谷信息产业基金，引进中国钛都等大项目103个，新增“新三板”挂牌公司10家。大力推进农旅深度融合发展，创成全国休闲农业与乡村旅游示范市，“丽水山耕”产品平均溢价率达33%。基本扭转丽水开发区工业经济持续下滑局面，丽缙五金科技产业园获批创建省级智能装备高新技术产业园区，智慧小镇科创园投入使用。建成“一机游丽水”综合服务平台，开展G20两千万旅游消费券大派送活动，缙云仙都景区获全市首张创5A“入场券”，旅游总收入、旅游投资分别增长25.8%、65.5%。服务业增加值增长8.7%、社会消费品零售总额增长11%，实现网络零售顺差63.18亿元。

　　（三）打好强统筹促融合组合拳。把大干快上交通项目作为统筹融合发展“先行官”，组织编制了项目总投资超千亿的美丽经济交通走廊建设总体规划。推动丽水机场项目获批，温武铁路线位争取工作进展顺利，高铁新站房投入使用，衢宁铁路顺利推进，金台铁路、瓯江航道整治工程开工建设，330国道莲缙段改建工程建成通车。着力打造最整洁、最礼让、最有序、最有爱心城市，创建全国文明城市年度测评成绩列全国第三。编制完成新一轮城市总体规划。继续推进美丽县城“六个一”示范项目建设，完成特色小镇投资202.9亿元，莲都区大港头镇、龙泉市上垟镇入选首批中国特色小镇。全面启动乡村建设转型综合试点，启动实施美丽林相建设20年规划，持续推进“六边三化三美”，打造美丽乡村风景线18条，8条道路获评“浙江最美绿化通道”。

　　（四）打好强民生促和谐组合拳。城乡居民人均可支配收入分别增长9.4%、9.7%，增幅均列全省首位，其中低收入农户人均可支配收入增长19.5%。深入推进城乡居民同类保障制度并轨整合及社会保障卡综合运用国家试点，医保个人账户“家庭共济”政策在全省率先落地，实现机关事业单位养老保险统一征缴与退休人员待遇统一发放。实现异地搬迁14276人，其中整村搬迁5554人、地质灾害隐患点避让搬迁7009人。交付保障性安居房4825套，完成农村危房改造3000户、城镇危险住宅房屋解危1031幢。建立市政府领导兼任教育特邀督学制度，高考上线率首次超过全省平均水平，启动了市区基础教育“三年攻坚”行动，松阳率先创成省基本教育现代化县。顺利实施全面两孩政策，区域人口健康信息平台上线运行，65岁以上老年人健康管理率达86.4%，新增97位百岁老人。牵头组建全国乡村春晚百县联盟，龙泉窑大窑—金村遗址入选“海上丝绸之路”申遗名单，成功举办丽水半程马拉松等重大赛事。实现了“平安夺金鼎”目标，圆满完成护航G20及世界互联网大会维稳安保任务。

　　（五）打好强改革促活力组合拳。坚持改革工作项目化推进，全年新争取9项国家级、31项省级改革试点。切实抓好国资营运公司市场化改革，将17家市本级国资营运公司整合为5大国有公司。立足于“功能区、集聚区和特区”的定位，完善了丽水开发区管理体制和运行机制。优化招商体制，狠抓招商项目落地。深入推进投融资改革，对政府和社会资本合作的项目融资实行银行综合金融服务政府采购，民间投资增速居全省首位。创建全国集体林业综合改革试验示范区，积极探索公益林补偿收益权质押融资。营改增改革试点全面推开。完成机关公务用车改革。实现市县乡三级农合联组建全覆盖。坚持改革开放互促共进，龙泉成为省外贸和实体商业转型升级试点示范县（市），云和成为产业集群跨境电子商务试点城市，全市外贸出口额增长6%。

　　（六）打好强效能促政风组合拳。强化主体责任，深入推进党风廉政建设和反腐败斗争，认真开展“两学一做”学习教育。出台“政风建设16条”，研究制定了部门职责管理办法、部门职责分工协商协调实施细则。开展“决战三季度”“冲刺四季度”专项行动，攻坚任务完成率达94.2%。在全省率先开展限额以下政府投资项目中介服务事项网上竞价，投资项目在线审批监管平台全网运行。紧盯项目抓效能，开展市区重点项目政策处理大会战、攻坚战，对PPP项目和道路交通、社会民生等领域重点项目推进情况实行“月报”，推动项目顺利落地、按时推进，全年完成固定资产投资841.65亿元，同比增长12%。严控“三公”经费，全市一般公共预算“三公”经费下降14.9%。

　　金杯银杯，不如百姓口碑。截至目前，我市有多项工作群众满意度列全省第一：生态环境质量公众满意度连续9年第一，食品安全公众满意率连续8年第一，新农村建设农民满意度连续5年第一，社会道德环境公众满意度连续4年第一，“五水共治”满意度、群众安全感满意率连续3年第一，民生改善满意度连续2年第一。这既是最好的褒奖，更是我们用心用力用情为民办实事的动力。

　　各位代表，这些年取得的成绩，都是在省委、省政府和市委坚强领导下，全市人民团结拼搏的结果。在此，我代表市人民政府，向全市人民和广大建设者，向人大代表、政协委员，各民主党派、工商联、人民团体和社会各界人士，向驻丽人民解放军、武警官兵和中央、省驻丽单位，向所有关心支持丽水发展的香港和澳门特别行政区同胞、台湾同胞、广大侨胞、海内外朋友们，致以崇高敬意和衷心感谢！

　　与此同时，我们也清醒看到发展中还存在不少问题：生态经济提速提质提效发展任务很重，亟需培育新的经济增长点，新旧发展动力转换不畅问题亟待破解；对照丽水特殊的生态屏障功能定位，生态保护和生态治理仍任重道远；城乡之间、县域之间发展不平衡问题依然存在，城乡居民尤其是困难群众持续增收基础还不稳固；基本公共服务供给与群众需求存在差距，防灾减灾等公共安全领域需要解决的问题还有不少。政府工作及政风建设中，一些部门打开“两山”转化通道的思想不够解放，一些干部在岗不在状态，甚至乱作为、不作为，有些改革举措和政策落实到不了位，“四风”问题也面临反弹压力。对此，我们一定高度重视，采取更加有力措施，认真加以解决。

　　二、今后五年发展的主要目标任务

　　科学把握历史方位，才有行稳致远的从容和自信。无论从时代特征、生态优势，还是现实基础、发展潜力看，丽水都已经具备从“两山”理论先行区向绿色发展示范区迈进的良好条件，正处于“变道超车”“穿越赶超”的机遇“窗口期”。

　　“窗口期”的机遇极为宝贵，抓住一次机遇，实现一次跨越；失去一次机遇，落伍一个时代。我们时刻要警醒：快人一步，领先一路；一步落后，步步落后。任何时候、任何环节、任何工作，都必须坚决贯彻习近平总书记“绿水青山就是金山银山，对丽水来说尤为如此”的重要嘱托，坚决扛起“与全省同步高水平全面建成小康社会，勇当绿色发展探路者和模范生”的历史使命，立足于下好先手棋、打好主动战，努力实现全面小康变“跟跑”为“齐跑”、绿色发展变“齐跑”为“领跑”。始终坚持“绿色发展、科学赶超、生态惠民”的发展主线，坚持“全域统筹、生态引领、城乡一体”的发展路径，坚持“改革推动、开放带动、创新驱动”的工作引领，坚持“生态底线、增收底线、平安底线”的工作要求，坚持“美丽环境、美丽经济、美好生活”的工作布局，充分体现“尤为如此”的示范和担当，努力让今后五年真正成为丽水发展的“黄金五年”。

　　根据市第四次党代会的战略部署，今后五年丽水发展的指导思想是：深入学习贯彻习近平总书记系列重要讲话精神，统筹推进“五位一体”总体布局，协调推进“四个全面”战略布局，以五大发展理念为指引，以“八八战略”为总纲，坚定不移走“绿水青山就是金山银山”绿色生态发展之路，加快推进“绿色发展、科学赶超、生态惠民”，着力打造美丽环境、发展美丽经济、创造美好生活，确保与全省同步高水平全面建成小康社会，加快建设“生态更美、产业更优、机制更活、百姓更富、社会文明程度更高”的美丽幸福新丽水，勇当绿色发展的探路者和模范生，为“浙江的今天”和“中国的明天”奉献更多丽水实践、丽水元素、丽水印记。

　　围绕实现市第四次党代会提出的奋斗目标，政府工作必须一以贯之深化细化具体化。尤其要通过实施“科学赶超五大行动”，确保生态环境状况指数稳居全省第一、空气质量和水环境质量全国全省领先，确保与全省同步高水平全面建成小康社会；实现全面小康人口、区域、领域全覆盖，基本公共服务常住人口全覆盖，社会保障法定参保人员基本全覆盖，实现国家生态县、国家卫生县城全覆盖，城镇截污纳管和农村生活污水处理、农村垃圾集中处理全覆盖，“六边三化三美”基本全覆盖；决不把脏乱差、污泥浊水、违法建筑、地质灾害重大隐患威胁带入全面小康，决不让一个困难群众在全面小康路上掉队，决不拖全省打造全面小康标杆省份的后腿。

　　今后五年发展的主要目标任务是：

　　——围绕生态保护第一责任，大力实施生态保护行动，建设全国生态文明先行示范区，构建天蓝地绿、山清水秀的生态丽水。基本建成全国生态文明先行示范区，生态环境公众满意度、生态文明总指数持续保持全省领先。严格执行新的环境功能区规划，严守生态红线，确保实现“两无”目标。统筹推进生态保护、生态修复、生态治理，森林覆盖率稳定在80%以上，林木蓄积量突破1亿立方米，永久基本农田不少于200万亩，工业危废全面实现无害化处理；2017年率先全域消除劣V类水，2018年全域创成“清三河”达标县，县级以上集中式饮用水源水质达标率100%；全域空气质量达到国家二级标准，市区空气优良率达到98%以上，全面消除中度以上污染天气。相关县（市）国家重点生态功能区、主体功能区建设试点取得明显成效，基本实现环境质量、重点污染源、生态状况监测全覆盖。

　　——围绕绿色发展第一要务，大力实施绿色发展行动，打造绿色发展“中国方案”丽水样板，构建产业绿化、结构优化的实力丽水。在全面完成“151”绿色发展行动计划基础上，形成较为完善的绿色产业、绿色产品供给体系，地区生产总值达到1700亿元，人均生产总值达到11000美元。以农旅融合为重点推进农业生态化、标准化、品牌化、电商化，创成省级生态循环农业示范市和农产品质量安全放心示范市，“丽水山耕”系列农产品年销售额突破100亿元，“丽水山居”农家乐民宿营业总收入突破100亿元。着力打造国家全域旅游示范区和国际生态旅游名城，大力培育生态旅游第一战略支柱产业，旅游总投资五年累计超1000亿元，期末实现旅游总收入1000亿元，旅游产业对GDP综合贡献率达到15%。着力打造全省生态工业示范市，把生态工业作为第一经济来培育，全面落实《丽水生态制造2025实施方案》，力争工业重点技改投资年均增长10%以上，战略性新兴产业、装备制造业、高新技术产业增加值占规上工业比重年均提高0.5个百分点，新一代信息技术融合应用达到全国先进水平，争创全国质量强市示范城市。着力打造浙西南生态服务业发展高地，累计完成服务业固定资产投资3000亿元，期末服务业增加值占GDP比重达到50%以上，确立高水平的“三二一”产业结构。

　　——围绕城乡统筹第一抓手，大力实施美丽丽水行动，打造“四美城乡”升级版，构建均衡协调、独具魅力的品质丽水。中心城市“一江双城三功能区”总体发展框架和“北居中闲南工”空间功能布局基本确立，完成城东和水南、城西、江滨“一片三区”开发，首位度进一步提高。市域“一心两轴四区四级”城镇空间结构基本形成，常住人口城市化率达到70%左右。深入实施“小县大城、小县名城、组团发展”战略，支持条件成熟的县适时启动撤县建市。全面推进大交通、大水利、大能源、大市政、大智慧“五大网络”建设，基本实现融入上海2小时交通圈、省域1小时交通圈，基本建成城乡1小时交通圈，县级以上城市防洪能力达到20-50年一遇标准，天然气管网实现“县县通”，城镇供水、燃气普及率均达到100%。全力推动美丽城乡建设，2017年，市本级确保创成全国文明城市；2019年，全面完成小城镇环境综合整治任务，实现县县基本无违建、建成“基本无违建市”；“十三五”期间，三分之二以上县（市）创成省级示范文明县城，成为“三美融合、主客共享”的江南山村旅居目的地。注重城乡建设内外兼修、古今融合，统筹抓好历史文化名城、名镇、名村和传统村落保护利用，进一步打响“丽水三宝”、巴比松油画、摄影等特色文化“金名片”。

　　——围绕改善民生第一追求，大力实施民生改善行动，不断增强群众获得感、幸福感，构建共建共享、安居乐业的幸福丽水。确保城乡居民收入可持续增长、收入增幅高于经济增幅、收入差距不断缩小，到2021年，城乡居民人均可支配收入分别达到51600元和26500元。坚决兜牢基本民生底线，确保省基本公共服务清单所列八大领域114个项目均达到全省均等化水平。不断完善覆盖城乡的就业创业和大社保体系，实现城镇“零就业”家庭动态归零，大力推进全民参保计划，每千名老年人拥有社会养老床位达到50张。做强农村集体经济，2019年底前全域消除集体经济薄弱村。打造全省民族工作高地，支持景宁建设畲族经济总部和文化总部，努力使民族乡镇总体发展不低于所在县（市、区）平均水平。努力与全省同步实现教育现代化，省义务教育标准化学校、等级幼儿园比例均达到90%以上，义务教育阶段学校基本实现小班化教学，支持丽水学院建设地方应用型大学，支持丽水职业技术学院打造现代高职教育品牌。建设“健康丽水”，人群主要健康指标达到或接近中高收入国家同等水平，人均期望寿命达到80岁。建设“文化强市”，确保文化体育事业主要指标居全省中上游、文化产业增加值占GDP比重达8%以上，全面实现“市有五馆、县有四馆”，全面完成二轮修志。强化基本公共安全，建成省级食品安全市，亿元生产总值生产安全事故死亡率下降30%以上、各类事故死亡人数下降15%以上，形成覆盖县乡村三级的避灾安置服务保障网。切实巩固“全国双拥模范城”创建成果，积极创建省级“军民融合创新示范区”。深入践行社会主义核心价值观，提高全社会法治化水平，基本实现社会治理体系和治理能力现代化。

　　——围绕改革创新第一动力，大力实施改革创新行动，不断激发经济社会发展新动能，构建动力澎湃、要素涌流的活力丽水。持续推进以全国生态文明先行示范区为龙头的国家级改革试点、以全域统筹绿色发展综合改革试验区为龙头的省级改革试点，基本完成丽水生态文明制度“四梁八柱”建设。探索建立绿色发展评价指标体系，以环境功能区规划为引领完善“多规合一”体系，积极推进资源环境市场化配置机制和投融资机制创新。打造对接多层次资本市场“丽水板块”，新增挂牌上市公司50家，新增直接融资超百亿。以大开放促进大发展，主动接轨“一带一路”、长江经济带和海西经济区，加快建设跨境电子商务产业园和建立华侨经济文化合作试验区，力争期末外贸进出口总额突破40亿美元、累计实际利用外资总额突破10亿美元。积极探索浙皖闽赣区域旅游发展合作机制，深化“山海协作”。加快步入创新驱动轨道，人才资源占人力资源总量比重达到26.2%以上，建成市级以上企业技术中心200家，R&D经费占GDP比重达到2.1%以上。

　　三、2017年政府工作主要安排

　　今年是实施“十三五”规划的重要一年、推进供给侧结构性改革的深化之年，也是新一届政府工作的起步之年。综合考虑发展大势与丽水实际，建议2017年全市经济社会发展主要预期目标为：地区生产总值增长7%以上；规模以上工业增加值增长7%以上；服务业增加值增长8%以上；旅游产业增加值增长10%以上；固定资产投资增长11%；社会消费品零售总额增长10%；出口总额保持适度增长；一般公共预算收入增长7.5%以上；城镇常住居民人均可支配收入增长8%；农村常住居民人均可支配收入增长9.5%；城镇新增就业岗位1.4万人，城镇登记失业率控制在3.5%以内；节能减排降碳指标完成或超额完成国家和省下达的目标任务，继续保持水环境质量全省最优、空气质量全国全省领先。

　　实现上述目标，需要奋发努力，在实际工作中争取更好结果。为此，今年政府工作必须坚持稳中求进工作总基调，主动把握经济发展新常态，大力推进供给侧结构性改革，持续深入打好“拆治归”转型升级系列组合拳，努力实现生态保护、生态发展、生态惠民的“多赢”。

　　（一）聚力生态文明抓示范

　　深化生态文明制度创新。深入研究重点生态功能区的“顶层设计”。严格落实领导干部自然资源资产离任审计和生态环境损害责任追究制度。做好饮用水水源保护管理条例、南明湖管理条例等生态立法基础工作。逐步建立和完善瓯江干支流梯级流量管理机制。出台生态破坏与环境污染突发事件应急预案，完善水气环境质量预测预警预报机制。积极推进环境监测监察执法垂直管理改革。

　　严格生态红线空间管控。强化生态保护红线、环境质量底线、资源利用上线和环境准入负面清单管理，建设环境监管网格化示范县。实现重点产业园区规划环评全覆盖，大力推进园区外企业整治入园，不能入园企业落实最严格监管措施，确保所有涉污企业达标排放，实现可管可治可控。完成工业固废综合处置场及危废焚烧处置扩建工程，实现危废处置能力“零缺口”。

　　推进生态环境综合治理。深入推进“五水共治”，进一步压实包括“小微水体”在内的河长制网格化管理责任，在全省率先全境剿灭劣V类水。完成所有城镇污水处理设施一级A排放达标改造，河道综合整治195公里。完成大规模畜禽养殖场粪污处理设施建设及散养生猪污染治理任务。大力推进“五气共治”，全面淘汰改造燃煤小锅（窑）炉。开展“净土”行动，构筑“舌尖上的安全”。处置25家僵尸企业，淘汰50家企业落后产能，促进500家以上“低小散”企业（作坊）完成整治任务。

　　（二）聚力产业培育抓突破

　　突出产业培育重点，以拓市场为导向推进“生态+”供给侧结构性改革。把握农业供给侧结构性改革机遇，在全面落实粮食安全市县长责任制的同时，以深化农旅融合为切入点，深入实施生态精品农业“912”工程，进一步打响“丽水山耕”“丽水山居”区域公用品牌。举办全市农产品包装设计大赛，不断提升特色农产品包装设计水平。新增“丽水山耕”农产品年销售额20亿元，新建“丽水山耕”合作基地1000家，新增农产品商标400件，新培育农产品旅游地商品生产经营主体和转化农产品旅游地商品各200 个，谋划建设一批旅游地商品购物中心。开展全域旅游示范区创建年活动，推进70%以上县（市、区）达到创建标准。加快建设20个农旅大融合产业集聚区、10条 “风情风景”旅游交通廊道，切实抓好5A景区创建，按照A级景区标准推进200个村景区化建设，力争创建3A以上景区20个，确保旅游投资增长20%以上。围绕打造高端装备制造、新材料、新能源等生态工业“千亿级产业”，力争市级工业投资项目库入库项目500项以上，组织实施100个以上战略性新兴产业项目。持续抓好“四换三名”和“个转企、小升规、规改股、股上市”，滚动实施小微企业三年成长计划，扶持一批品牌型、总部型、上市型龙头企业，培育一批超10亿元、超50亿元大企业，打造一批细分领域的“单打冠军”“隐形冠军”。办好中国“互联网+公共服务”创新大会，以“互联网+”“大数据+”“服务+”催生生态服务业新业态，实施100个以上服务业重点行业项目，完成电商经济“两中心一园区一基地”建设。

　　夯实产业发展平台。按照城市新区标准、“特色小镇”理念推进开发区（园区）改造提升，力争丽水开发区主要经济指标达到或超过全市平均水平，按照“百亿园区”目标抓好全市10个园区新增长点培育，新建标准厂房16万平方米以上。深入推进农业“两区一镇”建设，积极创建国家农业高新技术产业开发区。坚持以产业为核心、生态为基础、文化为灵魂、民间投资为动力，高起点推进特色小镇建设，争取实现县（市、区）省级特色小镇全覆盖。加快培育钛谷小镇、微纳小镇等先进制造业小镇，力争绿谷信息产业园入园企业达到150家。加快丽水海创园、华东药用植物园建设。实现无水港开港运营，完善外贸服务平台。

　　强化产业综合创新。推进产业政策创新，开展“精准服务企业、振兴实体经济”专项行动，持续抓好企业减负，出台做大做强外贸出口、总部经济、健康产业和现代建筑业、物流业、股权投资业等产业扶持政策。推进产业技术创新，深入实施高新技术企业和科技型中小微企业双倍增计划，新增高新技术企业38家、科技型中小微企业150家，规模以上工业新产品产值率达到32%以上。推进产业竞争力体系创新，坚持标准、质量、品牌强市，新增“浙江制造”标准5项以上，打造更有权威的地方生态产业标准，实施丽水老字号“振兴工程”，支持地理标志证明商标和集体商标做成做大做强。

　　（三）聚力项目推进抓投资

　　着眼发展后劲，狠抓有效投资。坚持把扩大有效投资、优化投资结构作为经济工作主抓手，确保重大基础设施项目、重大产业项目、重点工业技术改造、生态环保等4个领域投资均增长15%以上，力推全市重大前期项目、重点建设项目各100个以上，抓好30个左右PPP项目。加快“补短板”重大投资，兴起交通建设新高潮，确保丽水机场、衢丽铁路、水东综合客运枢纽等开工建设，加快衢宁铁路、金台铁路、瓯江航道整治工程建设，力推温武铁路、景文高速、庆寿高等级公路、庆景青公路等级提升和通用机场建设等项目前期，积极谋划城际轨道交通和市域铁路网；按照打造践行新时期中央治水工作新方针先行区目标，实施好水利“双百亿”工程，力争滩坑引水工程、缙云抽水蓄能电站开工建设，全面提速缙云潜明水库、松阳黄南水库、青田水利枢纽和瓯江独流入海治理等重大水利工程；确保金丽温输气管道工程建成通气，力争天然气“县县通”工程开工；加快新能源及送出通道建设。

　　坚持项目谋划、项目攻坚“两手硬”。全面打响项目谋划主动战。今年起，各县（市、区）五年内至少要谋划落实50亿元以上项目1个，每两年谋划落实20亿元以上项目1个，每年谋划落实10亿元以上项目1个、5亿元以上项目5个。切实打好项目攻坚整体战。完善项目审批“绿色通道”、政策处理攻坚常态化机制，探索设立基础设施投资基金，强化要素保障，确保项目推进“零障碍”。

　　（四）聚力招强引优抓招商

　　更加注重精准招商。坚持引资、引技、引智相结合，以产业招商为核心，强化浙商、外资、央企、民资、侨资“五资齐抓”。突出丽商、侨商回归，办好首届世界丽水人大会。强化招商统筹，科学编制“产业地图”和“招商地图”，实施挂图作战。深化产业链招商、“园中园”招商、专业招商等招商机制，大力开展资本招商，积极引进股权投资类企业。确保全年实际利用内资300亿元、外资2亿美元、浙商回归到位资金160亿元，引进大项目90个。

　　打造最优招商环境。坚持招商政策最优、服务最优、要素保障最优，研究完善差异化招商政策，强化“店小二”精准服务。健全与“一号工程”相适应的“全员、全程、全责”招商推进机制，建立党政“一把手”浙商回归工作季度专报制度，招商问题回应要“第一时间”、办理要“事不过夜”。

　　（五）聚力动能转换抓改革

　　以“最多跑一次”改革为推动，不断激发市场活力。按照走在全省前列、跑出丽水“加速度”的要求，从与群众和企业生产生活关联最紧密、受众最广泛的领域和事项做起，年内基本实现“最多跑一次是原则、跑多次是例外”改革目标。全面倒逼减权、放权、治权，加快形成覆盖行政许可、行政处罚、行政征收、行政裁决、行政服务等领域的“一次办结”机制，形成“双随机、一公开”“部门联合、随机抽查、按标监管”的“一次到位”监管机制，积极探索以“互联网+、标准化+”为助推器、“在线咨询、网上办理、证照快递送达”的便民服务事项“零上门”“一次也不用跑”运行机制。推进政府核准投资项目目录进一步“瘦身”，年内实现所有投资项目网上审批、核准、备案。探索政府购买审批中介服务。积极推动“区域能评、环评+区块能耗、环境标准”，逐步取代项目能评、环评。推进公共资源交易平台整合到位、高效联网。着力破解投融资瓶颈，探索产业基金市县联动机制，加快国资营运公司转型步伐，各县（市、区）旅游“三化”改革均要有实质性突破，持续抓好企业股改上市。

　　以打造全域统筹绿色发展综合改革试验区为推动，进一步打开“两山”转化新通道。总结近年来国家、省各项改革试点经验，强化改革系统集成效应，以“全域”为改革范围，以“统筹”为基本要求，以“综合”为主要方法，全市域谋划、全领域协同、全方位推进绿色发展综合改革。围绕生态资产资本化，深入推进林权、河道经营权改革，力争林权贷款余额超55亿元，探索建立小水电股权交易平台；深化与省股权交易中心合作，探索设立“丽水生态经济板”。加快县域经济体制综合改革，努力实现县县都有一项以上具有重大促进意义的国家级改革试点。联动推进农村产权、金融、扶贫改革，力求全国“两权”抵押贷款试点取得明显成效。启动国家气候适应型城市试点工作。完善市区共建共融共享机制。

　　（六）聚力城乡融合抓“三美”

　　统筹抓好城乡基础设施和治城、治乡基础性工作，打造美丽环境。围绕“修一条路、造一片景、活一方经济、富一方百姓”，在加快绿道建设的同时，每个县（市、区）至少各建设1条美丽经济交通走廊。围绕使城市既有“面子”、更有“里子”，三年内建设市区道路管线和地下管廊30公里，年内重点抓好桐岭路及中山街、丽阳街东段改造、绕城西线提升改造等项目，新实施36个老旧社区雨污分流改造，加快推进联城区块公共基础设施建设。以“砸锅卖铁”的决心推进地质灾害综合治理“大搬快治”，下决心用三年时间基本消除重大地质灾害隐患，年内完成避让搬迁项目162个、搬迁人口12552人，完成工程治理项目72个、解危人口5343人，新建、扩改建避灾安置点210个。深化“三改一拆”，打好城中村改造和治危拆违攻坚战，全市新开工城中村整体拆迁项目36个、5856户，综合整治城中村15个、5279户，完成全部D级危房和涉及公共安全的C级危房治理，完成拆违和“三改”面积各600万平方米。全面开展小城镇环境综合整治，确保完成50个乡镇（街道）的整治任务，重点打造20个示范小城镇。推进“坡地村镇”建设试点，实施城镇低效用地再开发1200亩，盘活存量建设用地3470亩。巩固提升农村污水治理设施运维率。基本实现农村生活垃圾集中收集处理全覆盖，分类处理建制村覆盖率达30%。

　　统筹抓好宜居、宜业、宜游丽水建设，发展美丽经济。以启动实施新一轮城市总规为契机，同步抓好城市有机更新和新兴产业发展，突出抓好“中闲”区块旅游休闲养生商务功能的培育。坚持美丽乡村和乡愁经济、美丽田园和养生经济“两结合”，谋划和建设一批“乡村创客”集聚区，大力发展民宿和“候鸟式”养生养老基地，支持有条件的乡村建设集循环农业、创意农业、农事体验于一体的田园综合体。坚持以美丽林相建设带动现代林业发展，实施美丽林相建设60.2万亩，栽植珍贵树种370万株，积极推广“一亩山万元钱”发展模式。

　　统筹抓好城乡文明创建，共创美好生活。突出城乡一体共建，抓实抓好市区新增停车泊位等20项重点创建工程，社区（城中村）和城郊结合部“六乱”专项整治等14项重点创建整治，深化社会主义核心价值观落细落小等30项重点创建载体，确保一举创成全国文明城市。结合信用丽水建设，启动“放心消费在丽水”行动，广泛创建放心市场、放心商店、放心网店、放心餐饮、放心景区、放心民宿。

　　（七）聚力民生福祉抓保障

　　全力抓好增收就业社保。把持续鼓起农民“钱袋子”作为头等民生大事，结合农业绿化、农村美化、农民转化，用好“老三宝”“新三宝”，确保农民收入增幅继续保持全省前列、力争全省第一。抓好改革活权促增收，深化“三权到人（户）、权跟人（户）走”改革，积极推进“三位一体”农合联建设。研究破解返乡下乡人员创业壁垒，加快“对外输出一人、致富一家”向“返乡创业一人、致富一方”转变。进一步巩固“消除4600”成果，加强因病、因学等“支出型贫困”家庭救助工作，推进保险助推扶贫试点全覆盖，加快光伏小康工程试点，实施集体经济薄弱村消除行动计划，完成农民异地搬迁10000人，其中整村搬迁不少于3000人。加强就业创业工作，健全就业困难人员兜底安置机制和政府购买公共就业服务机制，新增城镇就业1.4万人，城镇失业人员再就业6000人，其中就业困难人员2000人。落实好人才新政，大力引进紧缺实用人才，支持各类人才在丽创业。完善覆盖城乡更加公平更可持续的大社保体系，建立统一的城乡特困人员供养标准动态调整机制，建成全国社保卡综合运用示范基地。

　　加快推进基本公共服务均等化。持续抓好公共服务领域社会化改革，抓紧推进市科技馆、图书馆新馆、非遗馆、游泳馆等基础设施“补缺工程”。坚持教育优先发展，市县两级全面建立政府领导兼任教育特邀督学制度，逐步解决城区学校“大班额”和乡村学校“空心化”并存问题，力争龙泉、景宁、云和创成省基本教育现代化县，开工建设丽水学院松阳校区。研究制定《健康丽水2030行动纲要》。深化医疗、医药、医保联动改革，健全“双下沉、两提升”长效机制，培育30个基层区域医疗卫生服务中心，力争90%以上县（市）创成国家卫生县城。实现农村文化礼堂“乡村春晚”全覆盖，办好摄影文化节、第四届市运会。高度重视“老有所养”，基本实现城乡社区居家养老服务照料中心全覆盖，着力构建多渠道、多形式的老年综合服务体系。切实保障“住有所居”，基本建成保障性安居工程3653套、竣工3903套、交付入住1848套。加强困境儿童保障，加强残疾人权益保障。大力发展慈善和红十字事业。

　　努力打造“全国最平安城市”。推动平安丽水建设基础更扎实、机制更健全、人民群众安全感更高，争创“雪亮工程”国家级重点支持城市，实现乡镇（街道）基层治理体系“四个平台”建设全覆盖。确保食品药品安全，建立食用农产品产地准出与市场准入制度，争取全市95%的城区农贸市场建立溯源体系。深化安全生产体制机制创新，建立重点行业安全生产风险防控和隐患治理双重预防体系，加强防灾减灾和应急能力建设。扎实做好各类社会风险排查管控，切实防范金融风险。创新军民深度融合发展机制，扎实抓好国防动员、国防教育、双拥工作，大力支持国防和军队建设。

　　（八）聚力有为政府抓政风

　　把有为政府体现在勇于担当。强化政治意识、大局意识、核心意识、看齐意识，按照打造勇立绿色发展潮头丽水铁军的要求，扎实推进“干净干事创新业”作风建设专项活动，驰而不息抓政风。以干部“提神”促进发展提速提质提效，坚决摒弃袖手旁观的“看客”现象、按部就班的“蜗牛”速度，人人都要撸起袖子加油干、扑下身子抓落实！

　　把有为政府体现在善作善成。建立健全重点工作清单制度，加强政务督查和问责问效，并对部分重点工作、重点项目推进情况实行“周报”，紧要的节点性工作实行“日报”，力求天天有推进、周周有成效、事事有结果。坚持把对标一流、攻坚克难作为政风“试金石”，强化部门执行力建设，务求“三个在前”：工作时序要高效科学赶在前，只能提前，不能延后；急难险重要事不避难冲在前，只能抢着上，不能“躲猫猫”；“烫手山芋”要责无旁贷接在前，只能接好球、解好题，决不能只当“二传手”、凡事“往外推”。

　　把有为政府体现在干净干事。坚持把推进依法行政、划清权力边界作为干净干事之基，强化政府法治建设、信用建设，不打“擦边球”，不搞乱作为，构建“亲”“清”新型政商关系。坚持把自觉接受监督作为干净干事之要，自觉接受人大依法监督、政协民主监督和监察监督、司法监督，加强政府内部层级监督和审计监督，主动接受社会监督、舆论监督，依托“电视问政”等平台，进一步问出政府工作的问题思维、百姓思维、责任思维。坚持把严明纪律规矩作为干净干事之本，严格落实各级政府党组全面从严治党主体责任，推动中央八项规定精神风化俗成，坚决防止“四风”反弹回潮，坚决惩处各类腐败行为；严格落实财经纪律，以政府过“紧日子”换来群众和企业过“好日子”。

　　各位代表，人民政府为人民。政府的一切工作，都必须践行以人民为中心的发展思想。按照民生实事群众提、大家定的原则，今年的十方面民生实事经本次人代会票决确定后，我们将踏踏实实、全力办好。同时，将确保新增财力三分之二以上用于民生，从更多民生“关键小事”切入，加快让高水平全面小康走进丽水千家万户。

　　大时代呼唤大担当，新起点应有新作为。唯有干字当头，才能干出实实在在的美丽幸福新丽水。让我们更加紧密地团结在以习近平同志为核心的党中央周围，在省委、省政府和市委坚强领导下，不忘初心、继续前进，万众一心、苦干实干，以优异成绩迎接党的十九大和省第十四次党代会胜利召开，为丽水与全省同步高水平全面建成小康社会、勇当绿色发展的探路者和模范生而努力奋斗！
